# Supplementary material for: Surface Modification of Okara Cellulose Crystals with Phenolic Acids to Prepare Multifunction Emulsifier with Antioxidant Capacity and Lipolysis Retardation Effect
Source: Foods. 2024 Jan 5;13(2):184. doi: 10.3390/foods13020184 (PMC10813991; doi:10.3390/foods13020184)
Supplement: Supplementary file 1 [file foods-13-00184-s001.zip › foods-2785236-supplementary.pdf]

Table S1 Study on in vitro stability of the modified OC and digestion of the emulsions stabilized by modified OC

**GIT phase**	**Digestive fluids**<br>[composition; pH]	***In vitro* digestion**	
		**OC-G and OC-T**	**emulsions stabilized by the cellulosic materials or BSA**
**Oral**	**simulated saliva fluid (SSF)**<br>[KCl (15.1 mM), KH_2PO_4 (3.7 mM), $NaHCO_3$ (13.6 mM), $MgCl_2$ (0.15 mM), and $(NH_4)_2CO_3$ (0.06 mM); pH 7.0]	-	emulsion (5 mL) + SSF (4 mL) + α-amylase (750 units/mL)<br>↓<br>add $CaCl_2$ (0.1 M, 75 mL) and make up to 10 mL with DI water<br>↓ 37°C, 2 min<br>"bolus"
**Gastric**	**simulated gastric fluid (SGF)**<br>[KCl (6.9 mM), KH_2PO_4 (0.9 mM), $NaHCO_3$ (25 mM), NaCl (47.2 mM), $MgCl_2(H_2O)_6$ (0.1 mM), $(NH_4)_2CO_3$ (0.5 mM); pH 2.5]	modified OC + SGF (1:1, *wt/v*) + pepsins (25,000 units/mL)<br>↓ 37 °C, 2 h<br>← periodically take out a small aliquot of digestive fluids	bolus + SGF (7.5 mL) + $CaCl_2$ (0.1 M, 15 mL) + pepsin (25,000 units/mL)<br>↓<br>adjust to pH 3.0 using HCl (1 M) and make up to 20 mL with DI water<br>↓ 37 °C, 120 min<br>"chyme"
**Intestine**	**stimulated intestinal fluid (SIF)**<br>[KCl (6.8 mM), KH_2PO_4 (0.8 mM), $NaHCO_3$ (85 mM), NaCl (38.4 mM), and $MgCl_2(H_2O)_6$ (0.33 mM); pH 7.0]	add SIF (1:1, *wt/v*) +lipase (5,600 units/mL) + bile salts (160 mM, 2.5 mL)<br>↓<br>adjust to pH 7.0 using NaOH (1 M L^{-1})<br>↓ 37 °C, 4<br>← periodically take out a small aliquot of digestive fluids	chyme + SIF (6 mL) + pancreatic lipase (5,600 units/mL)<br>↓<br>add bile salt solution (160 mM, 2.5 mL) and $CaCl_2$ (0.1 M, 120 mL)<br>↓<br>adjust to pH 7.0 using NaOH (1 M) and make up to 40 mL using DI water<br>↓ 37 °C, 120 min<br>terminate the reaction by placing the mixture in an ice bath
**Determination**		TPC<br>DPPH radical scavenging ability	FFA content
